# Molecular modeling of LDLR aids interpretation of genomic variants

**DOI:** 10.1007/s00109-019-01755-3

**Published:** 2019-02-18

**Authors:** Eric W. Klee, Michael T. Zimmermann

**Affiliations:** 1 Department of Health Science Research, Division of Biomedical Statistics and Informatics, Mayo Clinic, Rochester, MN USA; 2 Center for Individualized Medicine, Mayo Clinic, Rochester, MN USA; 3 Bioinformatics Research and Development Laboratory, Genomic Sciences and Precision Medicine Center, Medical College of Wisconsin, Milwaukee, WI 53226-0509 USA

**Keywords:** Low-density lipoprotein receptor, Familial hypercholesterolemia, Molecular modeling, Genomic interpretation, Variant prioritization

## Abstract

**Abstract:**

Genetic variants in low-density lipoprotein receptor (LDLR) are known to cause familial hypercholesterolemia (FH), occurring in up to 1 in 200 people (Youngblom E. et al. 1993 and Nordestgaard BG et al. 34:3478–3490a, 2013) and leading to significant risk for heart disease. Clinical genomics testing using high-throughput sequencing is identifying novel genomic variants of uncertain significance (VUS) in individuals suspected of having FH, but for whom the causal link to the disease remains to be established (Nordestgaard BG et al. 34:3478–3490a, 2013). Unfortunately, experimental data about the atomic structure of the LDL binding domains of LDLR at extracellular pH does not exist. This leads to an inability to apply protein structure-based methods for assessing novel variants identified through genetic testing. Thus, the ambiguities in interpretation of LDLR variants are a barrier to achieving the expected clinical value for personalized genomics assays for management of FH. In this study, we integrated data from the literature and related cellular receptors to develop high-resolution models of full-length LDLR at extracellular conditions and use them to predict which VUS alter LDL binding. We believe that the functional effects of LDLR variants can be resolved using a combination of structural bioinformatics and functional assays, leading to a better correlation with clinical presentation. We have completed modeling of LDLR in two major physiologic conditions, generating detailed hypotheses for how each of the 1007 reported protein variants may affect function.

**Key messages:**

• Hundreds of variants are observed in the LDLR, but most lack interpretation.

• Molecular modeling is aided by biochemical knowledge.

• We generated context-specific 3D protein models of LDLR.

• Our models allowed mechanistic interpretation of many variants.

• We interpreted both rare and common genomic variants in their physiologic context.

• Effects of genomic variants are often context-specific.

**Electronic supplementary material:**

The online version of this article (10.1007/s00109-019-01755-3) contains supplementary material, which is available to authorized users.

## Introduction

Familial hypercholesterolemia (FH) is a genetic disorder causing high levels of low-density lipoprotein (LDL) cholesterol in patients beginning at birth and, due to lifelong exposure to high LDL levels, ultimately leading to heart disease and myocardial infarction at an unusually early age [1, 2]. It has a higher incidence in countries where genetic testing has become common, [3] indicating that it may be underdiagnosed. FH is caused by functional mutations in the LDL receptor (LDLR), its protein ligand (APOE or APOB), its recycling regulator (PCSK9), or its adaptor protein (LDLRAP1) that binds to the intracellular domain of LDLR. Deficiency of LDLR binding to LDL particles is a critical mechanism believed to underlie the majority of FH cases. Genomics sequencing to diagnose FH has led to the observation of many genomic variants altering amino acids within the LDL-binding domains of LDLR that lack any prior functional assessment. Without prior evidence of disease relevance, taking medical action based on these variants of uncertain significance (VUS) comes with risks for both the patient and medical practitioners. Patients may be treated for a genetic disease they do not have or fail to receive treatment for the one they do have. Lack of prior functional evidence is a barrier to the utilization of clinical genomics testing results. Therefore, in order to more fully leverage the data gathered from ongoing clinical genomics sequencing efforts, the clinical impact of these variants must be assessed.

A novel approach to understand how genetic variants may alter function includes accounting for the molecular structure of each protein domain. LDLR is composed of multiple domains and different domains mediate specific physiologic interactions. Class-A domains make direct contact with the protein components of LDL particles and their atomic structure is unknown for the extracellular conditions where receptor-particle encounters occur. Each class-A domain is about 40 amino acids long and has a calcium and pH-dependent structure [4, 5]. Experimental assays on the fifth class-A domain (LR5) have shown that the loss of calcium and acidic pH, characteristic of the endosomal environment, both contribute to LDL release by weakening the interaction with LR5 [4]. This is reflected in the 3D structure of LR5 around the calcium binding site, which interacts with protein ligands [4]. In this work, we integrated these and other data from the literature to generate a more comprehensive structural model for interpreting how genomic variants may alter any of the seven class-A domains at extracellular conditions.

The full molecular details of LDLR’s physiologic cycle have yet to be elucidated, but many states have been investigated using a wide variety of biochemical, spectroscopic, and bioinformatic approaches. LDLR undergoes a functional cycle from presentation on the cell surface to binding lipoprotein particles, internalization, endosomal release of lipoprotein particles, and recycling. Davis et al. showed, over 30 years ago, that deletion of LDLR class-B and EGF domains resulted in a receptor that was deficient in LDL binding and recycling but could still bind VLDL [6]. The following year, Esser et al. showed the necessary and additive role of certain class-A domains for binding each ligand and were the first to propose a higher order structure among the class-A domains [7], which was replicated soon after [8]. As the biochemical literature about LDLR grows, so too does the opportunity to enhance the interpretation of VUS using the resulting knowledge.

Establishing if a VUS leads to dysfunction of LDL binding will significantly inform clinical interpretation, thereby increasing diagnostic utility from clinical genomics sequencing. Contextualizing variant impact to LDLR cycle stage is clinically important as there are therapies that affect the system differently. While the overall domain architecture of the LDLR is established, the atomic structure at each stage in the cycle is not. Therefore, there is an opportunity and need to define the high-resolution structure of LDLR at multiple conditions, in order to better understand the physiologic impact of FH variants. Molecular modeling may provide additional information useful in determining the likely effect of each variant.

Current clinical paradigms use inheritance patterns, disease segregation, and repeated gene-phenotype observations to define causality or contribution of genomic variants to specific phenotypes [9, 10]. However, for rare disease patients, this can be significantly more challenging. To address this need, we can look towards mechanistic models to develop insight into variant effects on protein structure and function, thereby contributing to greater understanding and clinical interpretation. Experimental assessment of LDLR structure has revealed details of the endosomal stage of the LDLR cycle but has not elucidated details of LDL binding at extracellular conditions where LDL particles are recognized. In this study, we combine existing experimental data with computational structure modeling to generate high-resolution structural information accounting for conditions relevant to LDLR binding. The class-A domains directly interact with LDL particles and have the largest structural differences between the two conditions. The 464 amino acid variants observed within the class-A domains were evaluated using a combination of structure-based annotations and energetic calculations. This approach will provide mechanistic predictions for how each variant may alter LDLR structure, and thereby likelihood of altering binding to LDL particles.

## Results

**Fig. 1:**
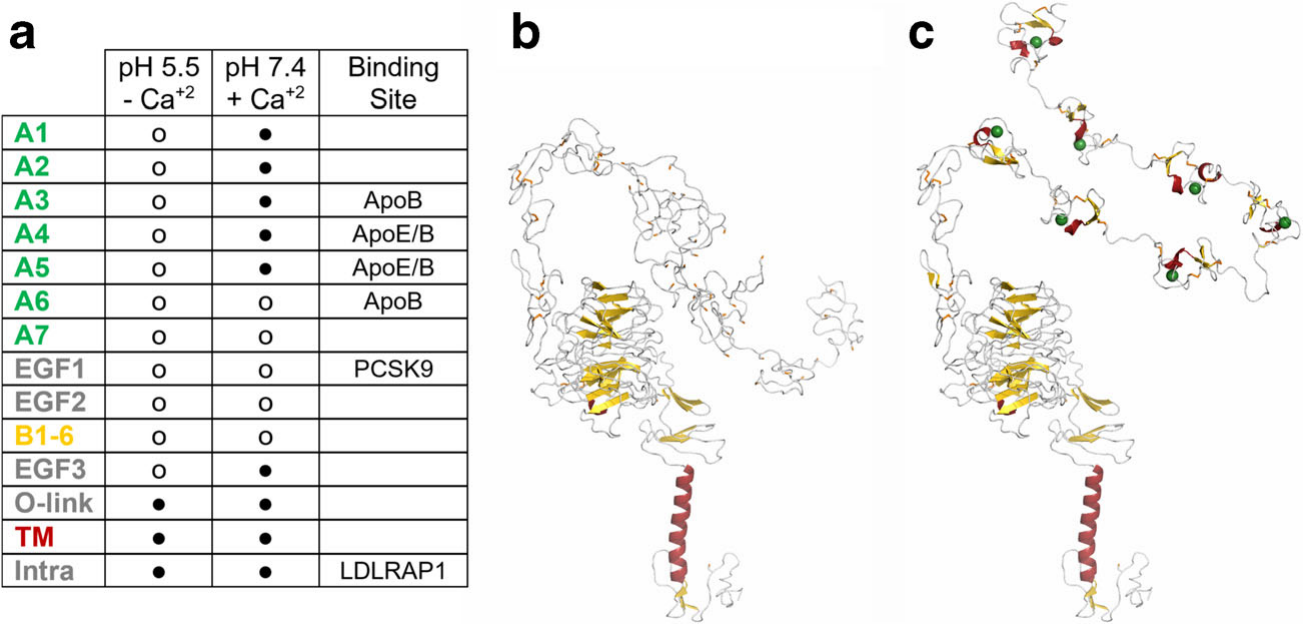
Structural model of LDLR at extracellular conditions generated by date integration and molecular modeling. **A** Each LDLR domain is available (o) or modeled by us (●). We have used molecular modeling to generate full-length LDLR structures at **B** endosomal and **C** extracellular conditions by leveraging available experimental data

**Fig. 2:**
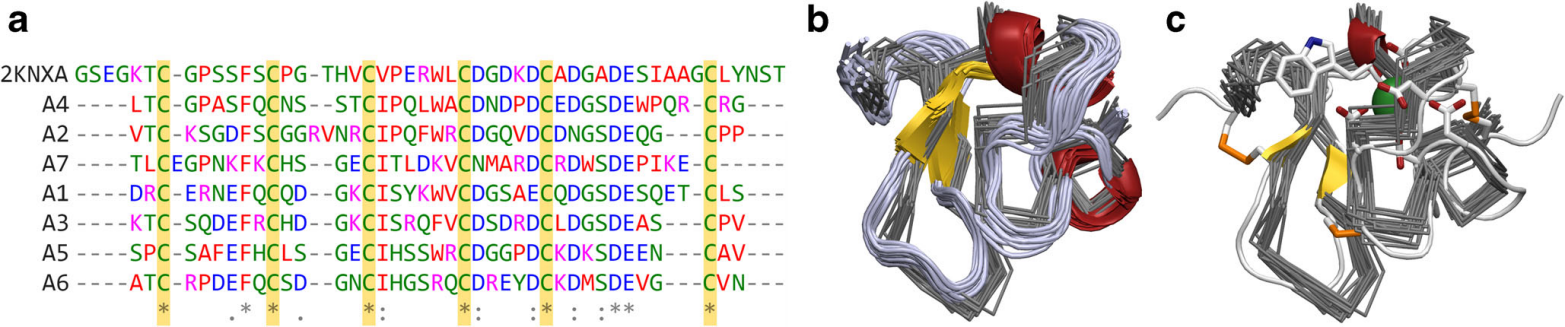
Conservation and modeling of LDLR Class-A domains. **A** The sequence alignment of class-A domains with the sequence of LRP-1 class-A domain, colored by amino acid type, shows the conservation of six cysteine residues and a pattern of acidic residues. These six cysteine residues form three disulfide bonds and the acidic residues form a binding pocket for Ca^+2^. **B** The backbone of LRP-1 class-A domain, solved by NMR, is shown in gray. All models from the NMR ensemble are shown. Superimposed and shown in cartoon representation, colored by secondary structure type, is LRP-1 class-A domain bound to the minimal peptide from ApoB. The overall fold is strikingly similar between bound and unbound conformations. **C** The backbone ribbons of LRP-1 are shown again, but now superimposed onto the fourth class-A domain of our extracellular LDLR model. The three disulfide bonds are shown in orange, Ca^+2^ green, and the residues interacting with the Ca^+2^ ion are shown in detail

We generate a model of the full-length LDLR at extracellular conditions (Fig. 1). As no full-length experimental LDLR protein structures at extracellular conditions exist, structural information for related domains from human paralogs was used. Each protein domain has a different level of existing data available from previous studies. Within the LDLR class-A domains, and the LRP-1 homolog, six cysteine residues, two acidic residues, and a single phenylalanine are the only conserved amino acids (Fig. 2A). Additionally, there is a pattern of negatively charged and polar amino acids. The cysteine residues are very likely to form three disulfide bonds and the pattern of acidic residues likely forms the binding site for Ca^+2^. Two examples of available experimental paralog structures include the LDLR-related protein 1 (LRP-1; Fig. 2B) and the single LDLR class-A domains, which have been experimentally investigated using NMR [11, 12]. These studies demonstrate the flexibility of each domain and the critical role of both Ca^+2^ binding and disulfide bonds to stabilize these domains. Additionally, these studies show that substrate binding has only minor effects on the structure, while changes to the environment, such as pH, result in significant structural alterations.

We assessed model quality for the class-A domains using multiple metrics. DPOE potential z-scores were less than z = − 2.5 for all class-A domains except for LR2 (z = − 1.9) and LR7 (z = − 1.7), indicating favorable energies compared to decoy models. The endosomal model has a high atomic clash score (z = 5.8), indicating many more clashes than experimental structures, while the extracellular model has a favorable clash score (z = − 0.3), indicative of an average experimental structure. The extracellular model only has 26% of residues involved in intramolecular hydrogen bonds, while our extracellular model has 48%. Finally, we considered dihedral angle scores. The endosomal model has 35% of residues in the Ramachandran core region, 82% in the allowed region, and outlier z-score of 11.8. The extracellular model has 70% of residues in the Ramachandran core region, 91% in the allowed region, and an outlier z-score of 2.8. Given that each class-A domain also contains disulfide bonds and Ca^+2^ coordination, we believe the model we generated for the extracellular state is of high quality and useful for annotating the potential effects of genomic variants.

Genomic variants were identified from the literature and public databases, mapped to our LDLR model, and observations regarding location within the protein model, and impacts on the computed structure made. Within the class-A domains, 58% of residues have identified variants in FH cases. For many of these variants, the clinical and/or functional effect is unknown, so detailed annotation using this structural modeling approach can provide valuable information for generating mechanistic hypotheses as to the variants’ effects.

There is a strong relationship between sequence conservation and the output of commonly used genomic sequence-based predictors. For example, there is a clear relationship between sequence conservation and classification by sequence-based methods such as PolyPhen-2 and MetaLR (*p* = 4.998 × 10^−4^). Structure-based ΔΔG_fold_ calculations across the entire protein are not correlated with sequence conservation (*p* = 0.971), but they are among class-A domains (*p* = 0.081). Overall, there is a strong correlation between ΔΔG_fold_ between models at extracellular and endosomal conditions (rho = 0.61), but the correlation is markedly different for the class-A domains (rho = 0.12). Thus, it is feasible that sequence-based predictors are less specific for highly conserved regions of LDLR, as has been previously identified in other systems [13]. However, structure-based annotations and calculations may address this limitation by providing results that are more specific for these regions.

**Fig. 3:**
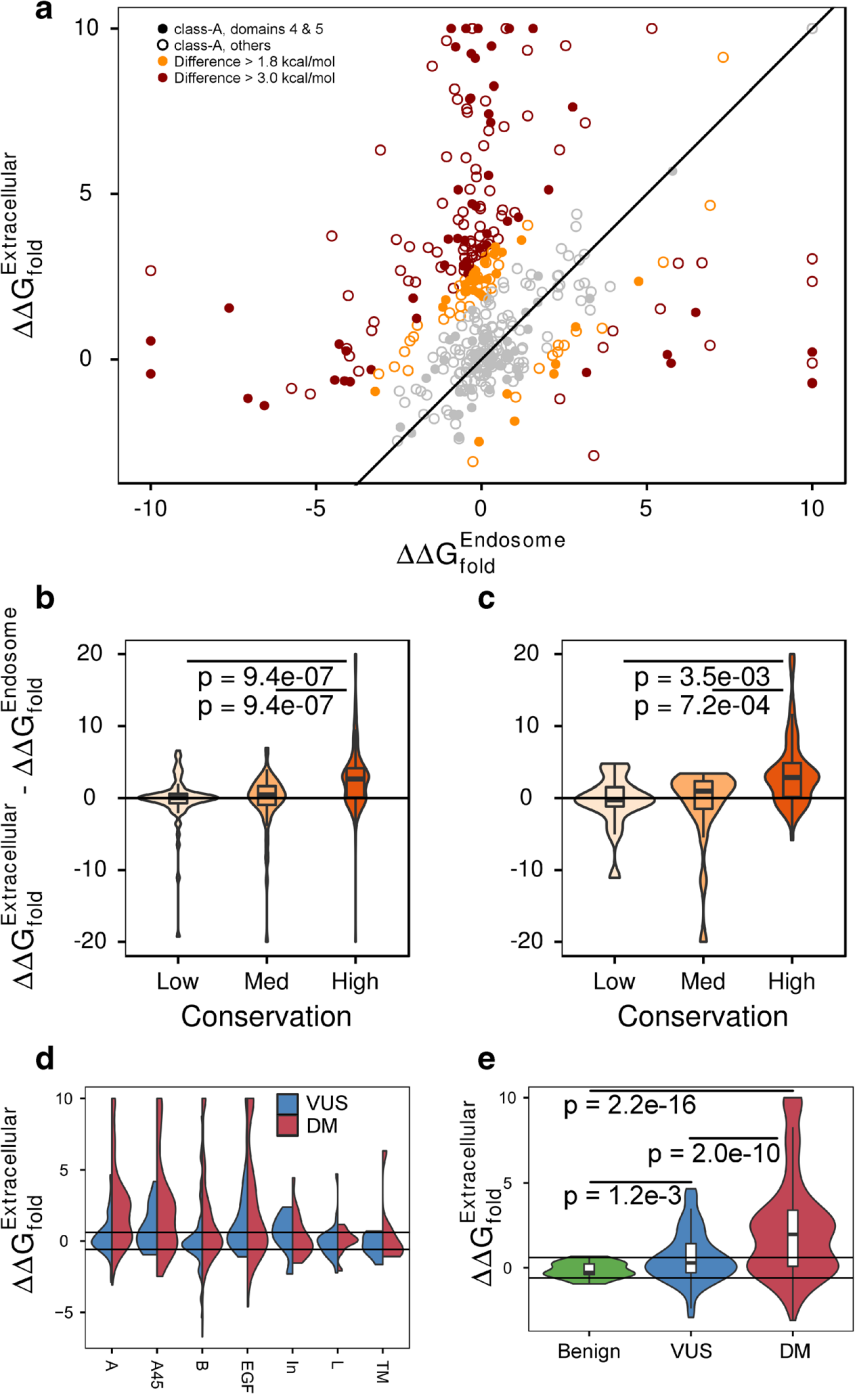
LDLR variants have context-specific effects. Each variant may confer significantly different effects on protein stability between endosomal and extracellular conditions. **A** Each data point represents a different LDLR variant. We evaluated 403 unique missense genomic variants observed in population (ExAC) or disease (ClinVar or HGMD) databases within the class-A domains. Symbols are filled in for the 128 variants from the fourth and fifth class-A domains. The line of equivalence is shown and variants colored gray if they exhibit a difference of less than 1.8 kcal/mol. The 57 (14%) of variants with a difference between 1.8 and 3.0 kcal/mol are colored orange, and the 119 (30%) variants with a difference greater than 3.0 kcal/mol colored red. **B** Across all class-A domains, there is a significant relationship between residue conservation and the difference in stability between conditions. **C** This relationship is present within the fourth and fifth class-A domains. **D** Across LDLR domains, missense variants in the class-A domains have the strongest separation in ΔΔG_fold_ between pathogenic variants and VUS. Horizontal lines mark 0.6 kcal/mol. Pathogenic missense variants in all extracellular domains are more likely to be destabilizing to the native structure compared to VUS. Many VUS in the fourth and fifth class-A and EGF domains are destabilizing. **E** For our extracellular model of class-A domains, there are strong differences between the distribution of ΔΔGfold among benign, VUS, and pathogenic variants. Not all pathogenic variants destabilize the conformation, but a significant fraction does. A smaller, but still significant proportion of VUS is destabilizing, but no benign variants are destabilizing

Using our models of LDLR in two physiologic states, we have annotated each variant with multiple mechanistic criteria: conserved at the protein sequence level, if it is likely (de)stabilizing, difference in ΔΔG_fold_ between the endosomal and extracellular models, if they affect necessary disulfide bonds, or if they are involved in the 3D coordination of Ca^+2^ ions (Table S1). Many variants not predicted to destabilize the endosomal structure were predicted to be highly destabilizing to the extracellular model (Fig. 3A). Patterns of evolutionary conservation are better described by the extracellular model than the endosomal model (Fig. 3B, C). Conserved residues make up the hydrophobic core of class-A domains that are denatured at high pH, or coordinate Ca^+2^ ions. Of the 403 variants observed within class-A domains, 374 affect one of the structurally-informed classes: 256 affect structurally conserved amino acids, 127 alter a disulfide bond, and 93 are likely to alter a Ca^+2^ binding site. Finally, we assessed differences in the distributions of ΔΔG_fold_ among benign, VUS, and pathogenic variants across domains (Fig. 3D). The class-A domains show the largest difference in distribution between variants of different disease association (Fig. 3E). Many VUS are also destabilizing or affect a specific structural role such as disulfide bond formation or Ca^+2^ coordination. Of the 24 literature-reported O-GalNAc modification sites in LDLR, nine are directly affected by genomic variants. These nine sites are affected by 12 different amino acid changes. From our structural model, five are stabilizing, six neutral, and one destabilizing to the protein structure. Thus, these multiple measures are additional lines of evidence for interpreting the likely functional implications of LDLR missense VUS.

## Methods

Human genetic variants in LDLR were downloaded from HGMD [14], ClinVar [15], and Leiden Open Variation Database (LOVD) [16, 17]. We gathered phenotypes from OMIM [18] and matched them with pathogenicity classifications from ClinVar, HGMD, and LOVD. For this work, we considered a missense variant to be pathogenic if it was labeled (likely) pathogenic in Clinvar, a disease mutation in HGMD, or of Association for Clinical Genetic Science (ACGS) class 4 or 5 in LOVD. We abbreviated pathogenic variants per the HGMD convention of DM for disease mutation. We considered a missense variant to be benign if it was labeled as (likely) benign in Clinvar or of ACGS class 1 or 2 in LOVD, and also lacked any of the criteria listed above for defining a variant as pathogenic.

Sequence and domain annotations of human LDLR were downloaded from UniProt accession number P01130-1 [19]. We used LRP-1 models (2nkx and 2nky) as templates to guide modeling of each of the 7 class-A LDLR domains. To do so, we generated a multiple sequence alignment [20], adjusted to ensure alignment of conserved cysteine residues that make conserved disulfide bonds. The pairwise residue equivalences to LRP-1 were used to make homology models in Modeler (version 9.17) [21, 22]. Each class-A domain model was computed independently. For each, multiple candidate models were generated and the model with minimum DOPE score chosen. These class-A domain models were bound to one another using a coarse-grained energy minimization [23] and assembled onto the remaining domains modeled using the endosomal experimental structure (1n7d) [24]. Our resulting model provided a basis for us to understand the effect of VUS under the extracellular conditions wherein LDLR binds its substrate. We used the model to identify residues involved in cysteine crosslinks and those likely to have a role in Ca^+2^ coordination. We considered changes in stability significant if they exceeded 0.6 kcal/mol and strongly altered if exceeding 1.8 kcal/mol. We used Foldx (version 4) [25] for computational mutagenesis and calculation of ΔΔG_fold_. Sites of post-translational modification were taken from the literature [26, 27] and PhosphositePlus database [28]. To evaluate model quality, we used DOPE z-scores and the VADAR webserver [29]. Conservation was assessed and mapped to our protein models using the ConSurf server [30] and 150 species’ sequences from UniRef90 aligned by ClustalW. Selected annotations were downloaded from dbNSFP [31]. Protein structures were visualized using PyMOL [32].

## Discussion

Current cardiovascular genetic testing is uncovering many genomic variants with uncertain clinical significance. Greater function and mechanistic resolution are required in order to properly treat patients with these variants. Previous studies by our lab [33, 34] and others [35, 36] have demonstrated that computational studies can generate novel data to strongly support the interpretation of variants identified from high-throughput sequencing and also to generate detailed mechanistic hypotheses for their underlying atomic mechanisms. When paired with detailed computational analysis, candidate mechanisms can be proposed at the atomic level to unify experimental observations with prior knowledge from the literature into a coherent mechanism of molecular dysfunction, driven by genetic variants. In this work, we develop computational and structure-based assessment to interpret the consequences of variants observed in LDLR, focusing on knowledge gained for the class-A domains.

We seek to extend the current clinical genomic sequencing paradigm, to include effects of LDLR protein structure and function changes in the interpretation of patient variants. Experimental structures of LDLR have been resolved, but at low resolution and for a limited number of physiologic conditions. A notable example is the lack of an experimentally determined LDLR structure at extracellular conditions where LDL particles are recognized. We generated new structure-based data for the class-A binding domains of LDLR and used them to predict each variant’s effect on domain stability. This data is relevant for interpreting the potential impact of variants observed in FH cases and likely more specific than sequence-based predictors. Further, we have aggregated multiple types of data from the literature to identify structure-based patters of conservation, cofactor binding, and post-translational modification across the receptor. Previous work has considered how genomic variants could alter the structure of LR5 or interaction with other proteins [37]. We have extended this concept to all class-A domains and integrated it with other data from the literature to provide a more comprehensive annotation for genomics data interpretation. Thus, our model of the extracellular conformation adds evidence for how missense variants may alter LDLR structure and function at a physiologic condition currently lacking experimental data.

It has been previously shown that multiple regions of LDLR are glycosylated. We identified that half of the glycosylation sites in LDLR are affected by genomic variants and stabilize the structure. Post-translational modifications often result in changing a protein’s conformation. Thus, it may be that genomic variants at these sites not only alter chemistry but lock the protein into one conformation. Further, of the 128 amino acids that are five or fewer residues away from a glycosylation site, 71 (55%) are affected by at least one missense genomic variant. Additionally, there are many genomic variants affecting residues near glycosylation sites, potentially modifying enzyme-binding motifs. Other motifs, such as the classic YWTD motif, have intra-molecular roles. The YWTD motif appears once for each class-B domain and makes up one of the beta-strands for each blade in the six-blade propeller fold; the beta-propeller fold is shared by multiple extracellular receptors that share the motif [38]. Previous low-resolution electron microscopy data of LDL particle structure identified a region of density that could be attributed to a bound receptor [39]. They placed one side of the class-B domain within this region of density. The class-B domain sequences interacting with LDL in their model have potential glycosylation sites that are not observed as glycosylated in multiple studies [26, 28]. While the class-A domains are regarded as the primary particle binding domains, it may be that certain regions of the class-B domains are protected from glycosylation through their interaction with other molecules. The interplay between glycosylation and genomic variants to modify intra- and inter-molecular features is an important dimension for future LDLR research.

Beyond the novel data from our model and aggregated from the literature, future studies may include additional environmental factors to be more informative for additional stages in the functional cycle. For example, experimental data indicates changes in the structure of LDL particles at endosomal pH [40], potentially altering receptor contacts. The cytoplasmic tail of LDLR forms oligomers regardless of the presence of LDL [41], and these data could enhance interpretation for residues within the cytoplasmic domain. In the future, additional experimental data, such as electron microscopy, for extracellular conditions may be generated. New experimentally derived structural data will be informative to the work presented here, and increase overall confidence in the hypotheses generated. However, we believe that modeling efforts such as these will remain informative as they enable in silico evaluation of patient-specific variants and the effect on LDLR structure and function. Further studies indicate that explicitly accounting for ligand, receptor, and environment may provide further mechanistic details across the LDLR functional cycle and the effects of missense variants.

Analysis of our full-length models of LDLR demonstrates that each variant may have a significantly different impact on the protein in different physiologically relevant conditions (Fig. 3). We have identified that many FH variants only have a strong effect at extracellular conditions, thus motivating the development of additional structural models and computational analyses to determine the most likely stage in the LDLR physiologic cycle that each variant may affect. Our model of LDLR under extracellular conditions provides clear interpretation of patterns of amino acid conservation; conserved residues typically fulfill specific structural roles in binding Ca^+2^ or contributing to the hydrophobic core of each class-A domain. Computational analyses afford the opportunity to predict effects in both pathogenic and protective directions, as has been clinically suggested for specific genetic variants [42] in LDLR. The current study has demonstrated additional knowledge that molecular modeling approaches can provide for interpreting the likely effects of coding variants affecting LDLR.

## Conclusion

To maximize the utility of genomics data and increase the impact of precision medicine, molecular models that can integrate the available experimental data to support functional interpretation of genomic variants are highly desirable. Establishing a molecular model typically yields immediate value because specific mechanistic hypotheses for the role of each amino acid becomes visually apparent. Then, it is much easier to hypothesize how those roles change due to genetic variation. The models we have generated in this study inform our understanding of the sequence-structure-function relationship for the LDLR—a critical protein in cholesterol metabolism. Additionally, they facilitate detailed hypothesis generation for the mechanisms by which genetic variants may alter LDLR—specifically, the extracellular state. Genomic variants may alter this state, or other states. Thus, additional studies could be made to further annotate which other states may be affected, and how, by genomic variants within this complex and dynamic protein. We believe that additional studies of the type we described here, complemented by functional assays, will yield mechanistic interpretation of each genomic variant and at high confidence.

## Electronic supplementary material

Table S1 (XLSX 112 kb)
